# A review of virtual planning software for guided implant surgery - data import and visualization, drill guide design and manufacturing

**DOI:** 10.1186/s12903-020-01208-1

**Published:** 2020-09-10

**Authors:** Florian Kernen, Jaap Kramer, Laura Wanner, Daniel Wismeijer, Katja Nelson, Tabea Flügge

**Affiliations:** 1 Department of Oral and Maxillofacial Surgery, Translational Implantology, Medical Center – University of Freiburg, Faculty of Medicine, University of Freiburg, Hugstetter Str. 55, 79106 Freiburg, Germany; 2 Department of Oral Implantology, Academisch Centrum Tandheelkunde Amsterdam (ACTA), Amsterdam, Netherlands; 3 Charité – Universitätsmedizin Berlin, corporate member of Freie Universität Berlin, Humboldt-Universität zu Berlin, and Berlin Institute of Health, Department of Oral and Maxillofacial Surgery, Berlin, Germany

**Keywords:** Guided implant surgery, Computer-assisted surgery, Computer-aided design, Virtual implant planning

## Abstract

**Background:**

Virtual implant planning systems integrate (cone beam-) computed tomography data to assess bone quantity and virtual models for the design of the implant-retained prosthesis and drill guides. Five commercially available systems for virtual implant planning were examined regarding the modalities of integration of radiographic data, virtual dental models and the design of drill guides for guided implant surgery. The purpose of this review was to describe the limitations of these available systems regarding the import of imaging data and the design and fabrication of a drill guide.

**Methods:**

The following software systems were examined regarding the import of imaging data and the export of the virtual implant planning for the design and fabrication of a drill guide with the help of two clinical situations requiring dental implant therapy: coDiagnostiX™, DentalWings, Canada (CDX); Simplant Pro™, Dentsply, Sweden (SIM); Smop™, Swissmeda, Switzerland (SMP); NobelClinician™, Nobel Biocare, Switzerland (NC); Implant Studio, 3Shape, Denmark (IST). Assessment criteria included data formats and management as well as the workflow for the design and production of drill guides.

**Results:**

All systems have a DICOM-interface (“Digital Imaging and Communication in Medicine”) for the import of radiographic data. Imaging artefacts could be reduced but not eliminated by manual data processing. The import of virtual dental models in a universal format (STL: Standard Tesselation Language) was possible with three systems; one system could only be used with a proprietary data format.

All systems display three-dimensional surface models or two-dimensional cross-sections with varying orientation for virtual implant planning. Computer aided design and manufacturing (CAD/CAM) of drill guides may be performed by the user with the help of default parameters or solely by the provider of the software and thus without the influence of the clinician.

**Conclusion:**

Data bases of commonly used implant systems are available in all tested software, however not all systems allow to plan and execute fully guided implant placement. An individual design and in-house manufacturing of the drill guide is only available in some software systems. However, at the time of publication most recent software versions showed flexibility in individual design and in-house manufacturing of drill guides.

## Background

Conventional implant planning is based on clinical examination and 2D radiographic imaging. The adoption of 3D radiographic imaging enables a more precise diagnosis of residual bone dimensions, the intrabony course of the inferior alveolar nerve and neighboring teeth [1, 2].

Individual patient 3D*-*imaging data is essential for virtual dental implant planning, computer aided design (CAD) and computer aided manufacturing (CAM) of a drill guide or implant-supported prosthesis. Anatomical data is derived from (cone beam) computed tomography (CT or CBCT) and optical scans of teeth and mucosa.

CBCT has a lower radiation dose (92–118 μSv) than CT (860 μSv) and is therefore often used for dental implant planning [3, 4]. Both CT and CBCT are stored in the universal format for “Digital Imaging and Communication in Medicine” (DICOM-format). Amongst imaging data, geometric and mathematical information, practical information such as acquisition details and settings are included in the DICOM file.

Volumetric imaging data is displayed in 2D cross-sectional images aligned to the prospective implant position. 3D surface models of CT or CBCT data are displayed using segmentation. Each voxel in the volumetric data set is assigned a grey value following its radiation attenuation, depending on the specific tissue characteristics. The display of a limited range of grey values enables the selective display of specific anatomical structures (segmentation).

CT or CBCT does not sufficiently display the tooth surface for the prosthetic set-up and for drill guide production. Especially in the presence of restorations, artifacts such as streaks and extinct areas occur [5]. Therefore, CT or CBCT scans and a virtual dental model obtained either from an intraoral optical scan or an extraoral scan of impressions or stone casts are aligned to each other prior to implant planning [6].

The data of intra– and extraoral optical scans are usually available in the universal stereolithography format (STL). This format contains geometric information of the surface [7]. Virtual dental models can be displayed in 2D along cross-sections and 3D to assess the mucosal surface from different viewpoints.

The process of aligning multiple imaging datasets with each other is defined as registration [8, 9]. Different procedures can be used to accomplish an accurate registration of CT or CBCT scans and virtual dental models: The tooth surface as a common structure displayed in both datasets may be used for registration. Custom and standardized reference markers (fiducial markers), respectively, can otherwise be introduced with a radiographic splint [10].

With standardized markers stored in the software, a single scan of the patient wearing the radiographic splint is performed (single scan protocol) [11, 12]. In the software, the stored reference marker is registered with the scanned image of the respective marker.

With custom markers a double scan protocol is used: after CT or CBCT acquisition of the patient with the radiographic splint, the radiographic splint alone is scanned [10, 13, 14]. The images of reference markers in both datasets are registered.

When using the tooth surface as a reference for registration, a splint with fiducial markers is not necessary [6, 15, 16]. The software uses an algorithm to register corresponding anatomical surfaces (automatic registration) or requires previous selection of corresponding areas by the user to initiate the registration process (semi-automatic registration). The accurate registration of CT or CBCT data and virtual models is crucial for a precise transfer of the prospective implant position to the surgical site [9].

After data import, segmentation and registration the prosthetic set-up and virtual implant position is planned. The prosthetic set-up combines the ideal position of implant-supported prosthesis and takes the abutment design with its emergence profile, morphology of the tooth, occlusal and proximal contacts into consideration. Using this information, implants can be virtually positioned in cross-sectional images and three-dimensional surface models reconstructed from the radiographic volume.

The design of a drill guide can vary depending on its function. It can either a) only guide the pilot drill (pilot guided) or b) guide every drill of the implant specific drill sequence (fully guided) [15, 17]. Additional to fully guided drilling, implant placement can be performed through the drill guide [11]. Guided protocols are preferred to complete free handed drilling and implant placement due to a higher accuracy of the implant position [14].

Drill guides may either be supported by the remaining teeth, the mucosa, directly by the bone surrounding the implant or by temporarily inserted mini implants [18, 19]. Especially in edentulous jaws with a mucosal support, the stability may be ameliorated with transitional screws or pins or temporary implants, securing the drill guide to the bone [20, 21].

In a fully digital workflow drill guides are virtually designed (CAD) and produced using computer-aided manufacturing (CAM). CAD/CAM is either performed by the software user or in a central production facility. The guides are milled from resin blanks [22, 23] or produced with an additive technique e.g. rapid prototyping [24]. In a combination of analog and digital techniques, drill guides are adapted from conventionally produced radiographic splints or produced on stone casts.

In this narrative review, the possibilities and limitations of five commercially available implant planning software systems are examined regarding the import of imaging data and the export of the virtual implant planning for the design and fabrication of a drill guide.

## Methods

The following commercially available virtual implant planning systems: coDiagnostiX, Version 9.9. (DentalWings, Canada) (CDX); Simplant Pro, Version 17 (Dentsply, Sweden) (SIM); Smop, Version 2.13. (Swissmeda, Switzerland) (SMP); NobelClinician, Version 2.4. (Nobel Biocare, Switzerland) (NC); ImplantStudio, Version 1.6.4.4, (3Shape, Denmark) (IST) were examined.

### Study design

The study design included the review of five different planning systems. Data of two patients with different indications for dental implant treatment were used to assess import and processing of imaging data for dental implant planning and drill guide production using CAD/CAM technology.

One patient had a missing single tooth in the region 21 (FDI), a fixed metal-ceramic prosthesis in the first quadrant and a cast post and core and metal-ceramic crown on the adjacent tooth (11 FDI). The second patient presented with a partially edentulous jaw with missing teeth in region 45–47 (FDI), a metal-ceramic crown on the adjacent tooth (44 FDI) and no other restorations in the lower jaw. CBCT data (3D Accuitomo, Scanora) and intraoral optical scans of the first patient (iTero, Cadent, Santa Clara, CA, US) as well as digitized stone casts (D250, 3Shape, Copenhagen, Denmark) of the second patient were available. CBCT data was stored in a DICOM format. Intraoral and extraoral scans and stone cast scans were available in the universal file format (STL). The above-mentioned virtual implant planning systems were evaluated by one examiner with defined assessment criteria as follows:

### Data acquisition and registration

Each system was examined regarding its options for the import of radiographic data (CT or CBCT) and virtual dental models. The availability of a proprietary scanner for intraoral scans or extraoral model and impression scans, respectively, and the data format specification for data import were assessed. Settings for the alignment of virtual dental models and radiographic data were evaluated regarding the use of single and double scan protocols and assistance of the system in the registration process (semi-automatic, automatic) (Table 1).

**Table 1:** Assessment criteria for data acquisition and registration of image data

**intraoral scans** **extraoral scans** **(CB-)CT**	importable data formats proprietary scanner available specifications (data format, image resolution)
**image registration**	single scan protocol using reference markers single scan protocol using tooth surface double scan protocol

### Visualization of imaging data

The visualization of CT or CBCT data was compared between the systems, regarding the options to select grey values for the display of distinct structures. Grey values for a segmented display of anatomy were selected manually or with pre-settings for certain structures (e.g. skin, bone, teeth). The selection of three-dimensional display options of CT or CBCT data, the availability of cross-sections as well as their setting and the orientation of models with the help of standard planes and views were assessed (Table 2).

**Table 2:** Assessment criteria for visualization of imaging data

**visualization of dental models**	2D display 3D display manual rotation and translation transparency selective display of initial situation and set-up
**visualization CT or CBCT data**	orthopantomographic view 2D cross sectional images 3D model rendering automatic and manual segmentation (grey value adjustment) individual editing of imaging artifacts bone density measurement

### CAD/CAM of drill guides

The spatial coordinates of the planned implant position were used for CAD/CAM of the drill guide. The possibilities for its design were examined for each system. The provided tools for fit, support and material thickness were documented. The options of in-house (individual) or centralized production of the drill guides were assessed (Table 3).

**Table 3:** Assessment criteria for automatic and manual drill guide design and production

**drill guide design and production**	supporting structures (teeth, bone, mucosa) guiding protocol (guided pilot drill, guided drill sequence, guided implant placement) export of drill guide design data set individual design and production of drill guide central design and production of drill guide

## Results

### Data acquisition and registration

**Fig. 1:**
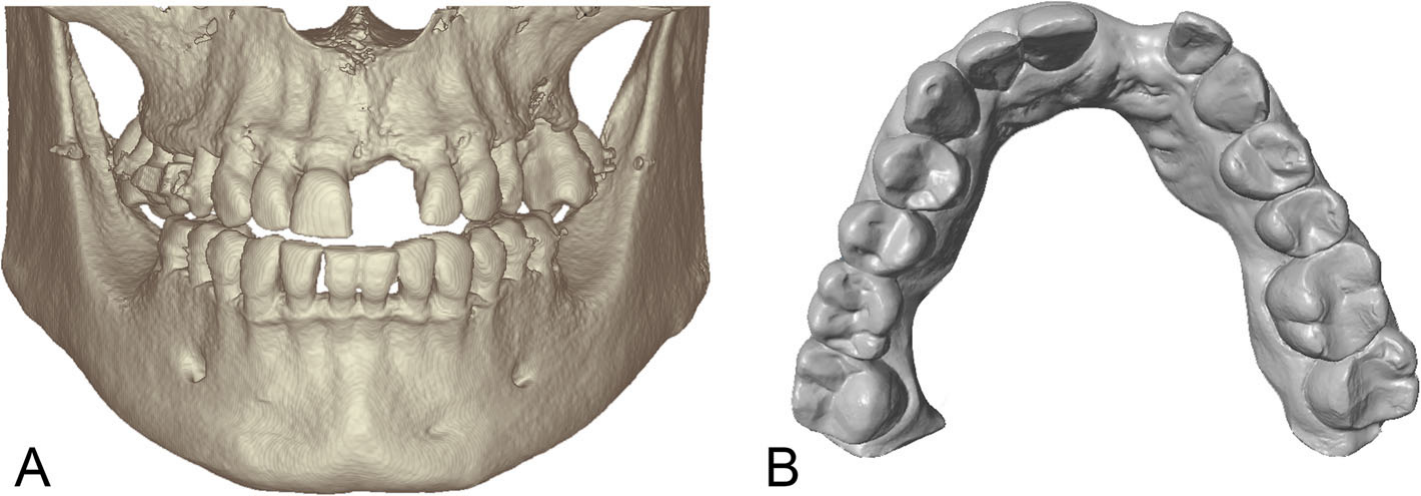
Display of data import options. All systems could import (CB-)CT data displaying the alveolar bone in the implant region (**a**) and virtual stone casts representing tooth and mucosa surfaces (**b**)

All examined systems used the universal DICOM format for CT or CBCT data import. The five examined systems allowed the import of scanning data in the universal STL-format (CDX, SIM, SMP, IST), while one system (IST) offered proprietary intraoral scanning and dental laboratory scanning technology (Trios, 3Shape). Intraoral scans acquired with Trios were displayed in Implant Studio™ with the texture and color of the teeth and mucosa. Another software (CDX) was linked with the software Cares (Dentalwings, Montreal, Canada) that provided a proprietary dental laboratory scanner for stone casts and dies. Both proprietary scanning systems provided virtual models as universal STL- files that might be used with any dental implant planning system (Fig. 1). One implant planning system (NC) exclusively imported model scans in a proprietary data format called NXA that could only be acquired with the proprietary dental laboratory scanner NobelProcera G2 (Nobel Biocare, Switzerland). The use of intraoral scans for implant planning was not possible with this system, as no proprietary intraoral scanner existed and no third-party intraoral scanner produced the NXA-data format.

The examined software systems offered different protocols for registration of CT or CBCT data and surface models. For single scans without reference markers, semiautomatic (CDX, SMP, SIM) or automatic (NC) registration algorithms using the tooth surface as a common structure in both images were applied. To initiate the semiautomatic registration process, the user selected corresponding areas on the surface segmented from CT or CBCT data and on the surface model of the teeth. In case of a visible deviation between the models, all examined software systems required the user to adjust the registration by manually moving the models in three-dimensional space (Table 4).

**Table 4:** Data import options for all the tested systems CDX (coDiagnostiX, Dentalwings); NC (NobelClinician, Nobel Biocare); SIM (Simplant, Dentsply); SMP (Smop, Swissmeda) and IST (Implant Studio, 3Shape)

	CDX	SIM	SMP	NC	IST
**(CB-)CT**					
DICOM	✓	✓	✓	✓	✓
proprietary CT or CBCT scanner	X	X	X	X	from 2017
**model scan**					
stl	✓	✓	✓	✓	✓
other	X	X	.ply format	.nxa format	.dcm format
proprietary model scanners	(✓)	X	X	Nobel Procera G2	✓
**intraoral scan**					
stl	✓	✓	✓	X	✓
other	X	X	X	X	.dcm format with texture
**image registration**					
double scan protocol	✓	✓	✓	✓	✓
single scan protocol with reference markers	✓	✓	✓	✓	✓
single scan without reference markers	✓	✓	✓	✓	✓
automatic registration	X	X	X	✓	X
semi-automatic registration	✓	✓	✓	✓	✓

### Visualization of imaging data

All examined systems provided tools for the selection of grey values for a selective display of anatomical structures. Bone and teeth could be visualized by selecting the appropriate range of grey values according to their density (Fig. 1). Default grey values for bone, teeth or soft tissues were given in all systems (CDX, SIM, SMP, NC, IST). Three systems allowed for selective editing of CT or CBCT data with virtual tools and separate masking and display of structures, e.g. neighboring teeth (CDX, SIM, NC). Two systems offered no individual tools for manual editing of three-dimensional models, but grey-value segmentation and separation of upper and lower jaw (SMP, IST).

Virtual models were displayed in 2D along cross-sectional views and as 3D surface models. A transparent 3D display served for better visibility of underlying bone. A color display of intraoral scans was only possible with one system (IST) that provided a proprietary intraoral scanner (Table 5).

**Table 5:** Results for visualization of CT or CBCT and dental models in tested software systems. (*Bone density measurements are based on Hounsfield units used for CT data and are not valid for CBCT data)

	CDX	SIM	SMP	NC	IST
**visualization of virtual stone casts**					
2D display	✓	✓	✓	✓	✓
3D display	✓	✓	✓	✓	✓
transparent display	✓	✓	✓	✓	✓
color display					✓
**visualization of CT or CBCT data**					
orthopantomographic view	✓	✓	✓	✓	✓
2D cross-sectional images	axial, transversal, tangential	axial, transversal, tangential	axial, transversal, tangential	axial, transversal, tangential	axial, transversal, tangential
3D model rendering	✓	✓	✓	✓	✓
automatic and manual segmentation	✓	✓	✓	✓	✓
Individual editing of imaging artifacts	✓	✓	X	✓	X
tool for bone density measurement*	✓	✓	X	X	✓

### CAD/CAM of drill guides

Drill guides could either be designed by the software user (CDX, IST) or in a central service and production center. SIM and NC only allowed drill guide design through a central service and production center. One software system offered both an individual and a central service for design and production of drill guides (SMP). The tested software systems allowed tooth-, bone- or mucosa-supported (CDX, SIM, SMP) or tooth- or mucosa-supported (NC, IST) designs. Drill guides for fully guided implant placement could be designed and produced for 11 (CDX), 16 (SMP), 26 (SIM), 45 (IST) implant systems, respectively. One system only offered guided-implant placement for proprietary implants and pilot drill for third party implant types (NC) (Table 6).

**Table 6:** CAD/CAM options and supporting surfaces for implant drill guides produced with the tested software systems

	CDX	SIM	SMP	NC	IST
**drill guide design (CAD) and production (CAM)**					
tooth support	✓	✓	✓	✓	✓
bone support	✓	✓	✓	X	X
mucosal support	✓	✓	✓	✓	✓
fully guided drill and implant insertion	✓	✓	✓	only proprietary implants	✓
implant systems for fully guided drill	11	26	16	1	45
export of drill guide design dataset for individual production	✓	X	✓	X	✓
individual design of drill guide	✓	X	✓	X	✓
central production of drill guide	✓	✓	✓	✓	✓

**Fig. 2:**
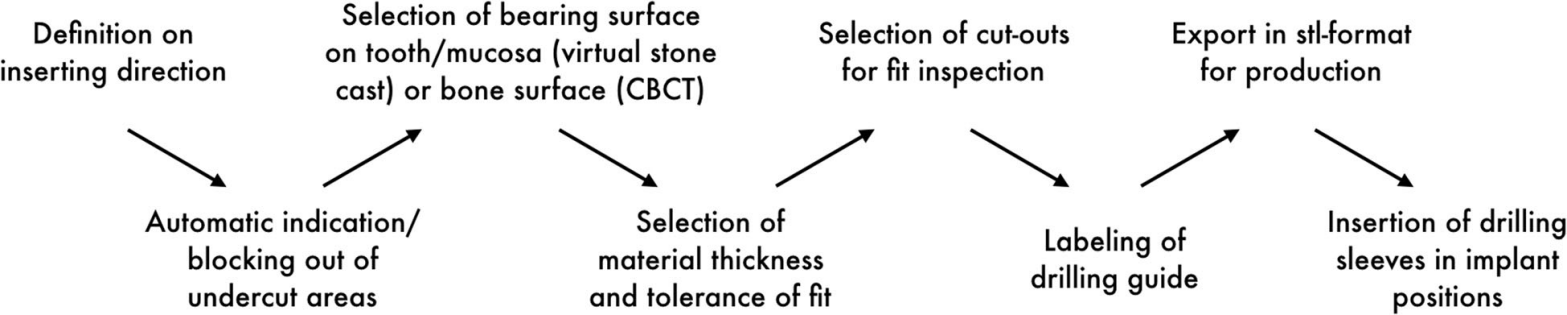
Workflow for drill guide design with software systems for individual drill guide design (CDX, SMP, IST)

The systems for an individual drill guide design (IST, CDX, SMP) allowed the user to define the bearing surface, the material thickness (IST, CDX), the tolerance between tooth surface and drill guide and the tolerance for inserting the drill sleeves, respectively (CDX). Undercut model surfaces were either virtually blocked out (CDX) or faded out (IST) automatically for control of intraoral seating. Drill sleeves were inserted according to the selected implant type. After defining the bearing surface, the software displayed a virtual model of the drill guide (Fig. 2). The bearing surface is chosen differently in each system. With NC and SIM the operator is able to choose the extension by selecting the teeth. In IST and CDX the extension of the guide can be defined based on markers which define the border of the guide. This gives more flexibility to the operator. In SMP the bearing surface is chosen in only selected areas due to the “open design” of the final guide. In this virtual display, windows could be inserted for intraoperative seating control of the guide (CDX, IST). An extended software module for drill guide design integrated in one software required the user to get a special training (SMP). The drill guide was composed of supporting and connecting elements with various diameters resulting in a skeletonized contact surface. Compared to the designs where the guide covers the selected area of the tooth (closed design) the SMP guides follow an “open frame” design. The supporting areas on a tooth are selected and connected in a tube-like design.

**Fig. 3:**
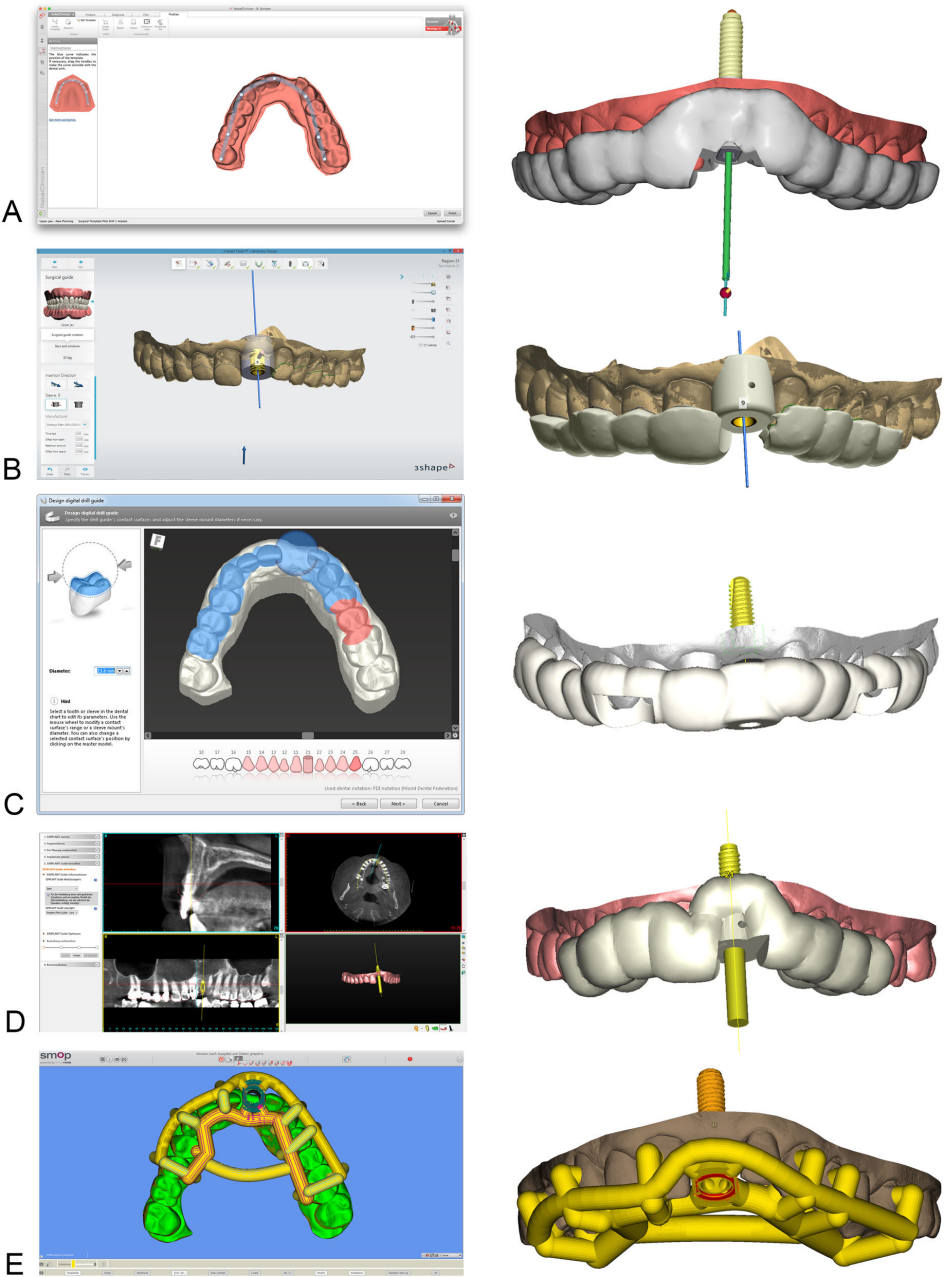
Design of implant drill guides in NobelClinician® (**a**), Implant Studio® (**b**), coDiagnostiX® (**c**), Simplant® (**d**) and SMOP® (**e**) by selecting the supporting surfaces

Alternatively, to the individual design of drill guides, the system displayed a virtual preview that could not (SMP) or slightly be modified by the user (NC). In this case the drill guide design was forwarded to a production center or a specialized dental laboratory for design and manufacturing (SMP). Individual labeling with patient initials or identification code on the drill guide surface was possible in IST, CDX, SMP and exported in an STL-format for in-office manufacturing (CDX, SMP, IST) or sent to a production center (Table 4). Design and display of the drill guides in the different software systems are shown in Fig. 3.

## Discussion

All tested implant planning systems used CT or CBCT DICOM data for bone diagnostics. None of the systems offered a proprietary CBCT scanner. To the knowledge of the authors, proprietary CBCT scanners are so far not available for any of the systems. Three-dimensional reconstructions and multiplanar cross-sections oriented along the alveolar process in the implant region were available in all systems to review important parameters for the implant position [25, 26].

With the clinical patient examples chosen in this study, imaging artefacts occurred distorting the tooth surface and bone volume. The examined software systems provided automatic segmentation of bone, teeth or soft tissues; however due to artifacts these default settings could not be used to display specific anatomical structures. Manual segmentation by limiting the window of grey values for the display of three-dimensional models was necessary and possible in all systems. Two systems did not offer tools to manually edit display of imaging data and two of the implant planning software provided tools for bone density measurement. Studies regarding grey values in CBCT data showed that they cannot be standardized and allocated to specific anatomical structures as in CT. Therefore, Hounsfield units used for interpretation of CT data are not applicable for CBCT data and bone density measurements in CBCT are not reliable [27].

The import, segmentation and pre-processing of radiographic data is crucial for the accurate transfer of the planned implant position to the surgical site. Radiographic data and virtual dental models are aligned with each other using either the tooth surface displayed both in CT or CBCT and in virtual dental models [9, 28] or with the help of reference markers in a radiographic splint [11, 12, 15, 29, 30]. Both workflows were available with the tested implant systems. Registration without a radiographic splint appears to be less time consuming as all examinations may be conducted without the preparation of a radiographic splint on a stone cast. However, misalignment between CT or CBCT and virtual models is known to occur after registration depending on the number of existing metal restorations [9].

The use of either an intraoral optical scan, or an impression or model scan, respectively, to produce a virtual dental model is freely selected by the user if the data is imported in the STL-format. One exception was found for NC, only importing virtual models in a proprietary data format generated by a system-specific model scanner. Intraoral scans including information on the color of teeth and intraoral soft tissue (Trios, 3Shape) were only compatible for IST planning software. The use in a third-party software was only possible after export to an stl-format that does not contain texture information. Therefore, implant planning with consideration of tissue quality is hitherto only possible with one system that includes a proprietary intraoral scanner with texture information (IST).

Intraoral optical scanning reduces the steps and therefore time expenditure to obtain virtual models [31, 32]*.* Besides the promising efficiency of intraoral scans, the accuracy of intraoral optical scanning is still not validated in vivo. In contrast, extraoral optical scanning of stone casts showed high accuracy (10 μm) [33]. However, the possible inaccuracy of a conventional intraoral impression and stone cast production are not included in the aforementioned studies. Inaccuracies of conventional intraoral impression should therefore be considered, when comparing the accuracy of intraoral optical impressions with extraoral model scans.

Depending on the used implant system either single steps or the full drill sequence and implant insertion is performed through the drill guide [10, 34–36]. The examined software systems allowed guided implant placement for a various number of integrated systems except for one implant system (NC) that only offered guided implant placement for its proprietary implant system. The selection of implant systems for which guided implant placement was provided was restricted and did not correspond to the number of systems offered for visualization. The selection of an implant planning software is therefore dependent on the specific implant systems used in the daily routine.

The support of the drill guide on teeth and mucosa, respectively, allows a more accurate transfer of the implant position than bone support [19, 37, 38]. The user could choose between the three bearing surfaces with exception of two systems (NC, IST), where no bone support was possible. Furthermore, pins or provisional implants could be inserted with all systems to help the fixation of the drill guide during surgery as suggested previously by other authors [39, 40]. Individual design of drill guides allowed the user to select bearing surfaces depending on each patient case. Whereas a closed guide design is suggested by most systems (NC, SIM, CDX, IST) an “open frame” design can be advantageous for more visibility, accessibility and less risk for interference with hard or soft tissue. Therefore, the insertion of windows in the closed design becomes important. With central design and production of drill guides, the user has to forward individual information regarding any specialties in the design prior to fabrication. The time consumption for personally designing and/or manufacturing of the drill guide and the cost of the software should be considered by the user, when using or choosing a virtual implant planning software. Two systems did not allow to individually plan nor individually fabricate the drill guides at the time of data collection (SIM, NC). To the knowledge of the authors, more recent versions of both software systems allowed individual production of the drill guide.

It has to be mentioned that user experience plays an important role in every CAD software. Depending on the user’s experience, their affinity to digital products the learning curve can vary. In summary, the authors find one planning software more intuitive than the other, which is very subjective. Before chosing a system it is recommended to test as many as possible to find a satisfactory product.

## Conclusions

Due to DICOM-interface, all implant systems could import radiographic data and three-dimensional reconstructions or two-dimensional cross-sections. When dental radiographic imaging is impaired by streaking artifacts, e.g. metallic restorations, all software systems allowed to manually segment the CT or CBCT data and CDX. SIM and NC offered a reduction of imaging artifacts by manual processing of data. Virtual implant planning systems allow either an individual design and production of drill guides or referral to a production facility where the fabrication is centralized. The construction of the drill guides result in similar designs except for the open design with SMP. Depending on the selected virtual planning software a varying selection of implant types may be planned for guided implant surgery. Further studies should investigate the time consumption associated with the use of the software to evaluate the relation of time and cost. Outsourcing parts of the planning process can become a viable option in the future and should be taken into consideration.

## Data Availability

The datasets generated and analysed during the current study are not publicly available since they are not encrypted but are available from the corresponding author on reasonable request.

## Abbreviations

CAD: Computer Aided Design
CAM: Computer Aided Manufacturing
CBCT: Cone beam computed tomography
CDX: coDiagnostiX
DICOM: Digital Imaging and Communication in Medicine
IST: Implant Studio
NC: NobelClinician
SIM: Simplant
SMP: Smop
STL: Standard Tesselation Language
